# Chemotherapy promotes tumour cell hybridization in vivo

**DOI:** 10.1007/s13277-015-4337-7

**Published:** 2015-11-05

**Authors:** Bingyu Yan, Jianguo Wang, Li Liu

**Affiliations:** State Key Laboratory of Biocontrol, College of Ecology and Evolution, Sun Yat-sen University, #135 Xinggang West, Guangzhou, 510275 China

**Keywords:** Cell fusion, In vivo, Chemotherapy resistance, Poor prognosis

## Abstract

Spontaneous cell-cell fusion has been recognized to be an important mechanism for tissue and organ development and repair. In cancer, cell fusion is critically involved in tumourigenesis, metastasis and drug resistance, as illustrated by in vitro experiments. However, there has been no direct detection of tumour cell fusion or hybridization in an in vivo tumour environment, and the features of hybridized cells under selective pressures, such as chemotherapy, are unknown. Here, we expressed two fluorescent marker proteins in the human breast cancer cell line SKBR3 to detect tumour cell hybridization in vivo and performed a xenograft chemotherapy experiment in mice to evaluate the chemotherapeutic response of the hybrids. The mice treated by epirubicin showed that chemotherapy promoted tumour cell hybridization in vivo, which elicited the production of more hybrids in the outer section of the tumour. These results provide the first in vivo evidence of tumour cell fusion and indicate that chemotherapy may contribute to a poor prognosis by enriching for fused cells, which are more malignant. It is therefore necessary to reassess chemotherapy strategies.

**Electronic supplementary material:**

The online version of this article (doi:10.1007/s13277-015-4337-7) contains supplementary material, which is available to authorized users.

## Introduction

Spontaneous cell–cell fusion is essential in many important biological processes, such as fertilization, placenta development, bone and muscle formation, tissue regeneration, immune response, tissue repair and stem cell differentiation [1–4].

In the field of cancer research, Otto Aichel first hypothesized that leucocyte–tumour cell fusion or hybridization could lead to the emergence of malignant cells and neoplasms in 1911 [5]. Cell–cell fusion has been recognized as an important biological process in cancer evolution since Pawelek et al. discovered that normal cell–tumour cell fusion is involved in tumour initiation and metastasis [6, 7] and Miller FR et al. demonstrated that in vitro tumour–tumour cell hybrids were more malignant and chemoresistant [8, 9]. In addition, spontaneous cell–cell fusion may occur during chronic inflammation, which is related to neoplasia [10]. Thus, spontaneous cell–cell fusion could play important roles in many aspects of tumour progression, such as the generation of cancer stem cells and the acquisition of metastatic potential or multidrug resistance [11, 12]. Chemotherapy has been utilized to treat cancer for many years, and this approach kills most of the tumour cells, but patients still have a poor prognosis because of drug resistance caused by intratumoural heterogeneity and other factors [13, 14]. Multiple ways can lead to this heterogeneity. Stochastic genetic or epigenetic changes are well-established mechanisms involving intrinsic differences among cancer cells [15, 16]. Genetic and phenotypic heterogeneity are major causes of variations in chemotherapeutic responses [17]. Cell–cell fusion can lead to heterogeneity [18]. Cells with different genetic backgrounds that hybridize in vivo may enrich for multiple important mutations and then develop drug-resistant subclones, as has been reported based on in vitro experiments [8, 19]. Moreover, except mutations occurred in stem cells, epithelial–mesenchymal transition (EMT) and de-differentiation of differentiated cells or cells in the late progenitor stage, spontaneous cell–cell fusion can produce recurrence cancer stem cells (rCSCs) after prognosis as well, which could increase the patient’s risk of experiencing recurrent or metastatic disease [12, 20].

Therefore, spontaneous cell–cell fusion has a considerable impact on chemoresistance, metastasis and prognosis in cancer treatment. In vitro experiments can provide preliminary information on cell fusion during tumour evolution, but these experiments do not recapitulate in vivo tumour evolution under selective pressures, such as chemotherapy. Furthermore, there have been only a few reports on in vivo tumour cell fusion in human cancer and the corresponding chemotherapeutic response, which is the key evidence that illustrates the important roles of cell fusion in tumour evolution [6, 19, 21, 22] and its critical impact on cancer treatment.

Here, we introduced enhanced green fluorescent protein (EGFP) and red fluorescent protein (mCherry) transgenes into the SKBR3 cell line to perform xenograft experiments in Balb/c-nu mice and treated these xenograft mice with epirubicin chemotherapy to construct an in vivo chemotherapy tumour evolution model that better mimics cancer evolution in patients. Cell hybrids, which express dual fluorescence in a single cell, were detected by fluorescence-activated cell sorting (FACS). Furthermore, chemotherapy was found to promote tumour cell hybridization in vivo by generating more hybrids in the outer section of the tumour. These results provide evidence to support previous studies on cell–cell fusion in vitro [6, 8, 19] and provoke safety concerns regarding chemotherapy.

## Materials and methods

### Transgenic cell line construction and cell culture

We replaced the EGFP gene in pll-3.7 with the mCherry gene and inserted a puromycin resistance gene downstream to construct a plasmid called pll-mCherry-puro. We used pll-3.7 with a neomycin resistance gene downstream of the EGFP ORF to construct pll-GFP-neo. We transfected the pll-EGFP-neo/pSPAX2/pMD2.G and pll-mCherry-puro/pSPAX2/pMD2.G lentivirus packaging systems into HEK293T cells according to the Lipofectamine® LTX DNA Transfection Reagent (Invitrogen) Protocol. We harvested the EGFP virus medium and the mCherry virus medium 48 h after transfection by aspirating HEK293T cell culture medium and then used a 0.22-μm Millipore filter to filter the virus-containing medium.

The SKBR3 human breast carcinoma cell line was plated at 20∼30 % confluence the day before infection. We individually mixed the filtered EGFP virus medium and the filtered mCherry virus medium with equal volumes of fresh basic Dulbecco’s modified Eagle’s medium (DMEM) (Gibco) supplemented with 10 % foetal bovine serum (FBS) and then added the two mixtures to two different wells containing SKBR3 cells. Forty-eight hours after virus transfection, we screened the cell lines using the corresponding screening medium: DMEM basic supplemented with 10 % FBS and puromycin (Sigma-Aldrich) at a final concentration of 2 μg/ml for mCherry-puro cell lines and DMEM basic supplemented with 10 % FBS and neomycin (Sigma-Aldrich) at a final concentration of 0.8 μg/ml for EGFP-neo cell lines. The two prepared cell lines were named SK3R (mCherry) and SK3G (EGFP) based on their fluorescent protein expression. We examined these two cell lines and observed that more than 99 % of the transgenic cells expressed their respective fluorescent protein as evidence by fluorescence-activated cell sorting (Fig S1).

The transgenic SK3R and SK3G cells were cultured in DMEM basic (Gibco) supplemented with 10 % FBS and 100 U/ml penicillin at 37 °C in a humidified atmosphere of 5 % CO_2_ prior to the xenograft experiments.

### Xenograft and drug treatment

All the animal studies were conducted in accordance with the protocol approved by the Institutional Animal Care and Use Committee (IACUC) of Sun Yat-sen University.

The cultured SK3R and SK3G cells were trypsinized, counted and mixed in equal numbers. The cell mixture (2 × 10^6^) was diluted in 70 μl of PBS mixed with 70 μl of Matrigel™ (BD) and injected into the mammary fat pads (MFP) of eight 4-week-old Balb/c-nude mice. The tumour xenografts were observable in the MFP 4 days after injection. When the diameter of the xenograft tumour reached ∼10 mm, we separated the mice into the chemotherapy treatment group (three mice) and the null control group (three mice).

The mice in the chemotherapy group were treated with 8 g/kg epirubicin (Pfizer, China) by tail vein injection [23]. The null control group received an injection of an equal volume of saline.

### Tumour size calculation, harvest and lysis

When the tumours appeared in the MFP of the mice, we used a vernier calliper to measure the long diameter (*a*) and the short diameter (*b*) of the tumour to calculate the tumour size using the equation *V* = *ab* ^2^/2 [24]. We calculated the tumour size during tumour expansion, drug treatment and drug withdrawal.

One and a half weeks after chemotherapy treatment, the mice were killed and the tumours were harvested. Each tumour was cut into the outer section (∼2 mm thickness) and the inner section (∼10 mm diameter). An entire half of the tumour was retained as another section. Before they were digested with collagenase, these xenograft tumour sections were cut up into small pieces (∼1 mm^3^) and then minced completely using sterile blades. To obtain single-cell suspensions, the resulting tumour pieces were then mixed with ultra-pure collagenase III (Sigma-Aldrich) in medium 199 (Gibco) (200 U/ml) and incubated at 37 °C with shaking at 150 rpm for 3–4 h [25]. The cells were washed twice with HBSS and filtered using 100-μm BD Falcon™ cell strainers to prepare for FACS.

### Cell staining, fluorescence-activated cell sorting and fluorescence microscopy

Before FACS, the sorting gates were determined for SKBR3 (gate B), SK3R (gate A) and SK3G (gate C) cells (Fig S1). We used 620/29 nm channel to detect red fluorescence and 529/28 nm channel to detect green fluorescence in the experiment, and auto compensation in the MoFlo XDP program was used. The tumour single-cell suspensions were resuspended in PBS and sorted on a MoFlo XDP Cell Sorter (Beckman Coulter, USA). Cells (∼10^6^) were analysed for each tumour. We purified the cells in gate P, which represents the positive cells with double fluorescence.

The purified dual-labelled cells and gated out cells were stained with Hoechst 33342 (Life Technologies), washed twice with PBS and examined by FACS for DNA content. The sorted cells were examined by fluorescence microscopy (EVOS® FL Imaging System).

## Results

### Spontaneous cell fusion between tumour cells is involved in tumour evolution in vivo

To detect tumour cell hybridization in vivo, two sublines of the human breast tumour cell line SKBR3 were constructed by introducing two transgenes, each expressing a fluorescent protein, by lentivirus: SK3R and SKBR3 cells labelled with mCherry and SK3G and SKBR3 cells labelled with EGFP. A 1:1 mixture of SK3R and SK3G cells in PBS was mixed with 50 % (*v*/*v*) Matrigel and injected s.c. into the mammary fat pad (MFP) of 4-week-old Balb/c-nu nude mice. Four days after the injection, tumours were observed in the MFP of these mice. When the tumour reached ∼15 mm in diameter, the tumour from one mouse was minced and digested into a single-cell suspension, and the cells were analysed by FACS.

**Fig. 1:**
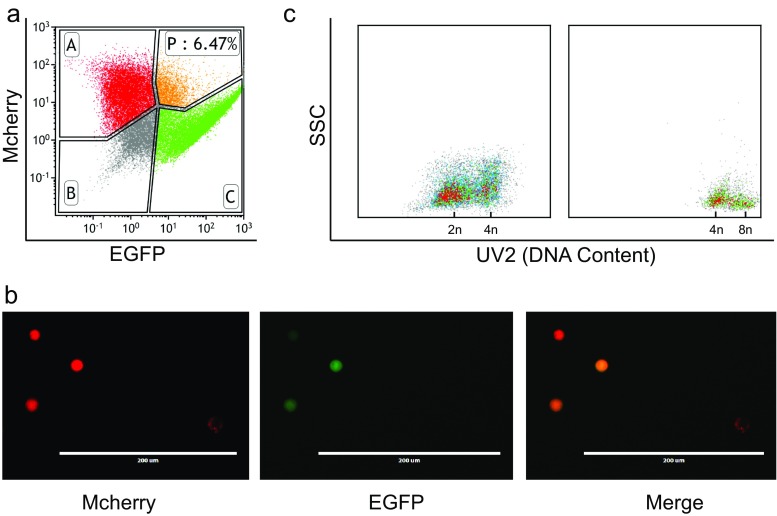
Spontaneous tumour cell hybridization in vivo. **a** Detection of EGFP–mCherry-hybridized cells by fluorescence-activated cell sorting (FACS). Gate P represents cells expressing both fluorescent markers, and the proportion is labelled in the *bracket*. **b** Fluorescence microscopy. *Left panel*, mCherry channel; *mid-panel*, EGFP channel; *right panel*, merged channel. The fused cells have both mCherry and EGFP fluorescence. **c** The DNA content of the non-fused cells (*left*) and fused cells (*right*). Cells were stained with Hoechst 33342 and sorted by FACS

Cells containing both fluorescent signals were defined as hybrid cells. As shown in Fig. 1a, 6.47 % of the tumour cells were in gate P, indicating that these cells simultaneously expressed EGFP and mCherry. To confirm this observation, the cells in gate P were sorted and re-analysed by fluorescent microscopy. These cells were indeed dual-labelled (Fig. 1b). In addition, the sorted cells were stained with Hoechst 33342 and analysed by FACS. The sorted cells had twofold or more DNA content compared to the single-labelled cells (Fig. 1c). There was no other source of double-labelled cells except tumour cell hybridization during tumour growth. Therefore, SCF of tumour cells did occur in vivo at a higher frequency (6.47 %) than predicted (1 %) [26].

### Tumour cell hybrids were enriched in the tumour after epirubicin chemotherapy in vivo

**Fig. 2:**
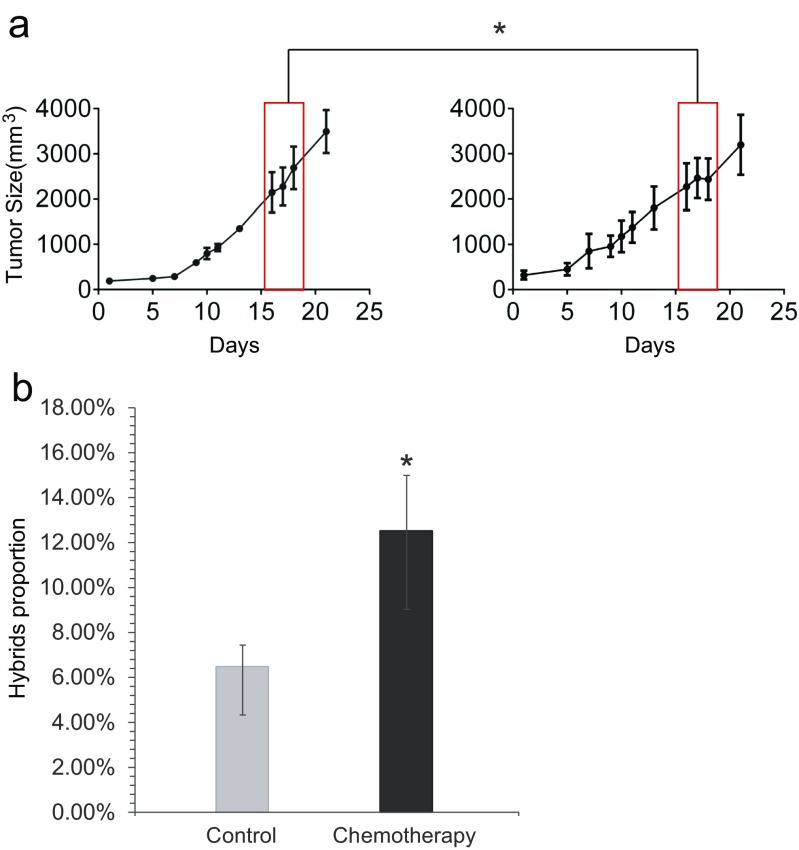
Chemotherapy enriches tumour cell hybrids in vivo after drug treatment. **a** Tumour size at various stages: tumour initiation, tumour progression, drug treatment and drug withdrawal. Drug treatment occurred on the 15th day after tumour appearance. The *left panel* represents the non-chemotherapy control group; these tumours expanded in a nearly exponential manner. The *right panel* represents the chemotherapy group; after chemotherapy, these tumours initially expanded more slowly than before, but after a week, they started expanding as they did before chemotherapy as the drug’s effect diminished. The *y*-*axis* represents tumour volume (mm^3^), and the *x*-*axis* represents days after tumour appearance (*p < 0.05). **b** Proportion of hybridized cells between the non-chemotherapy and chemotherapy groups. The chemotherapy group had more hybrids because their population was enriched by the drug treatment

Many in vitro cell fusion experiments have suggested that tumour cell hybrids can be more drug-resistant and more metastatic than parental cells [8, 9]. To assess the chemotherapeutic response of the hybrids in vivo, we separated the xenografted mice into the chemotherapy group and the non-chemotherapy control group. When the tumours reached ∼10 mm in diameter (15 days), the mice in the chemotherapy group were treated with epirubicin via tail vein injection on the 16th day. After drug treatment, the growth rate of the tumours in the chemotherapy group initially was four times slower than in the non-chemotherapy group (*p* < 0.05), but then it expanded faster compared to the non-chemotherapy group, though there was no significant difference (Fig. 2a, Table S1). Tumours in both groups were minced and digested into single-cell suspensions, and the cells were analysed by FACS. The tumours in the non-chemotherapy control group had a population of 6.1 ± 1.6 % fused cells, and those from the chemotherapy group had a significantly increased proportion of hybrid cells (12.2 ± 3.0 %, *p* < 0.05) (Fig. 2b). Thus, chemotherapy appeared to promote tumour cell hybridization in vivo, potentially because the tumour cell hybrids are less sensitive to chemotherapy than the non-hybridized tumour cells.

### Heterogeneity of the hybrids in the tumour during tumour expansion under selection

**Fig. 3:**
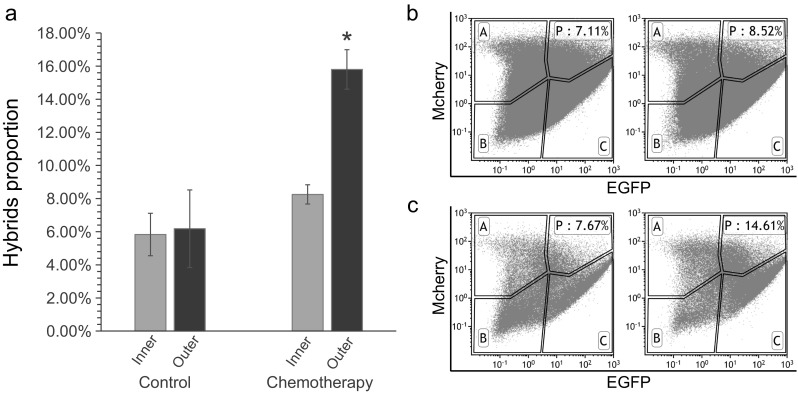
Heterogeneity of cell–cell fusion during tumour expansion after chemotherapy. **a** The proportion of hybridized cells in different parts of the tumour in the non-chemotherapy and chemotherapy groups. There was no significant difference between the outer and inner sections in the non-chemotherapy group. However, in the chemotherapy group, the outer section of the tumours contained a higher proportion of hybrids than did the inner section because of the more rapid expansion of hybrid cells compared to non-fused cells after chemotherapy (*p < 0.05). **b** FACS analysis of tumour cells from mice in the non-chemotherapy group (*left*, inner section of the tumour; *right*, outer section of the tumour). **c** FACS analysis of tumour cells from mice in the chemotherapy group (*left*, inner section of the tumour; *right*, outer section of the tumour)

To investigate the distribution of the hybrids in the tumour in vivo, tumours were divided into outer (∼2 mm thickness) and inner (∼10 mm diameter) sections and analysed (Fig. 3). There was no significant difference in the hybridization frequency between the outer and inner sections in the non-chemotherapy group (Fig. 3a, b); that is, the distribution of spontaneous cell–cell fusion in tumours is homogeneous in their natural state. By contrast, in the chemotherapy group, more hybridized cells were found in the outer section (15.8 ± 1.2 %) than in the inner section (8.3 ± 0.6 %) of the tumours (Fig. 3a, c). Chemotherapy apparently changed the distribution of spontaneous cell–cell fusion in tumours. The hybridization frequency in the inner section was comparable in both groups, whereas that in the outer section was significantly different between the two groups (Fig. 3a). A reasonable explanation is as follows: the tumour cell hybrids, which are less sensitive to chemotherapy, could survive at a higher proportion during chemotherapy and promote tumour expansion after drug withdrawal (Fig. 2a); meanwhile, the inner section was less affected by chemotherapy because there are relatively fewer vessels in this section. Another more attractive speculation is that chemotherapy may facilitate spontaneous cell–cell fusion of tumour cells.

## Discussion

Because of intratumoural heterogeneity, different cells have different traits based on their own genetic background [13, 14]. In the Darwinian evolutionary view, tumours live as a population in their microenvironment [27, 28] and experience certain selective pressures, such as chemotherapy. During tumour evolution, some cells develop “driver” mutations that facilitate a subclone to survive and gradually obtain more malignant traits (e.g. metastasis and drug resistance) [29]. It is conceptually difficult for a differentiated cell to obtain these abilities through de novo mutations, which are generated during a random and long evolution process in a single cell [30]. Until now, it was thought that the expansion and evolution of a tumour was only an asexual process [31]. However, if cell fusion events analogous to sexual reproduction occur during cancer evolution, the resulting hybrids with more DNA or genome copies could harbour more mutations, which is the underlying cause of the observed refractory phenotype. Thus, individual cells with different genetic backgrounds could combine or exchange their genetic material, such as drug resistance- or metastasis-associated mutations. Lu et al. reported that hybrid cells formed by the spontaneous fusion of two sister subpopulations were more clinically aggressive than parental cells [32]. This result suggested that cell–cell fusion in tumours may represent a sexual reproduction method for adapting to the altered microenvironment. Tumour evolution should be considered not only at the genomic level but also at the dynamic population level with hybridization between different cells. Tumour cell hybridization is an effective way for the population to evolve and survive under diverse selective pressures. This study demonstrated that cell–cell fusion could occur during tumourigenesis and progression in vivo (Fig. 3a, b).

Cell–cell fusion can permanently change the genetic background of hybridized cells [33]. Furthermore, it can lead to epigenetic changes; many experiments have shown that fusion of an enucleated cell and a somatic cell nucleus can reprogramme the somatic nucleus into a pluripotent state [34]. Hybridization between tumour cells and stem cells has been recognized as one of the sources of cancer stem cells [35].

As we discovered, the fused cells survived and became enriched during drug treatment in vivo (Fig. 3a). The increase in the relative proportion of hybridized cells indicated that the hybrids were less sensitive to chemotherapy than were the other cells, which is consistent with in vitro experiments [36].

Moreover, hybrid cells were enriched in the outer section of the tumour, and the tumour continued to expand under chemotherapy (Figs. 2a and 3a), indicating that after drug withdrawal, the tumour expanded faster due to the proliferation of these drug-insensitive hybrids. This proliferation despite chemotherapy is a sign of poor prognosis. At the late stage of cancer, these hybrids are in the outer section of the tumour. If they harbour metastatic mutations or hybridize with metastatic cells, the chemoresistant metastatic cells could create a more complicated obstacle for cancer treatment.

The selective pressure on cell–cell fusion can expand to the division of hybridized multiploid cells; during asymmetric cell division, certain genes or chromosomes that enhance drug resistance or other malignant traits may be retained [19, 32]. Therefore, it is urgently necessary to understand the mechanisms of cell–cell fusion in cancer evolution. Candidate drugs that target the cell–cell fusion process could reduce the increased malignant potential generated during this process. In this study, we detected tumour cell fusion and its distribution in the whole tumour by the cell proportion directly. However, it needs more specific and detailed researches by other methods like immunohistochemistry in the future.

## Electronic supplementary material

Below is the link to the electronic supplementary material.

Figure S1 Control gates for FACS. a. Gate B: SKBR3 cells expressing no fluorescent proteins (negative control gate). b. Gate C: SKBR3 cells only expressing EGFP (EGFP control gate). c. Gate A: SKBR3 cells only expressing mCherry (mCherry control gate). d. Gate P: SKBR3 cells expressing both EGFP and mCherry in each cell (positive control gate). e. A mixture of SKBR3 cells expressing no fluorescent protein, EGFP or mCherry. These data were used to calculate the false positive rate. f. A mixture of SKBR3 cells expressing no fluorescent protein, EGFP, mCherry or both EGFP and mCherry. These data were used to calculate the discrimination ability of FACS. (PDF 1136 kb)

Table S1 (PDF 64 kb)

## Abbreviations

FACS: Fluorescence-activated cell sorting
