# Supplementary figures and images for: Chemotherapy promotes tumour cell hybridization in vivo
Source: Tumour Biol. 2015 Nov 5;37(4):5025–30. doi: 10.1007/s13277-015-4337-7 (PMC4844647; doi:10.1007/s13277-015-4337-7)

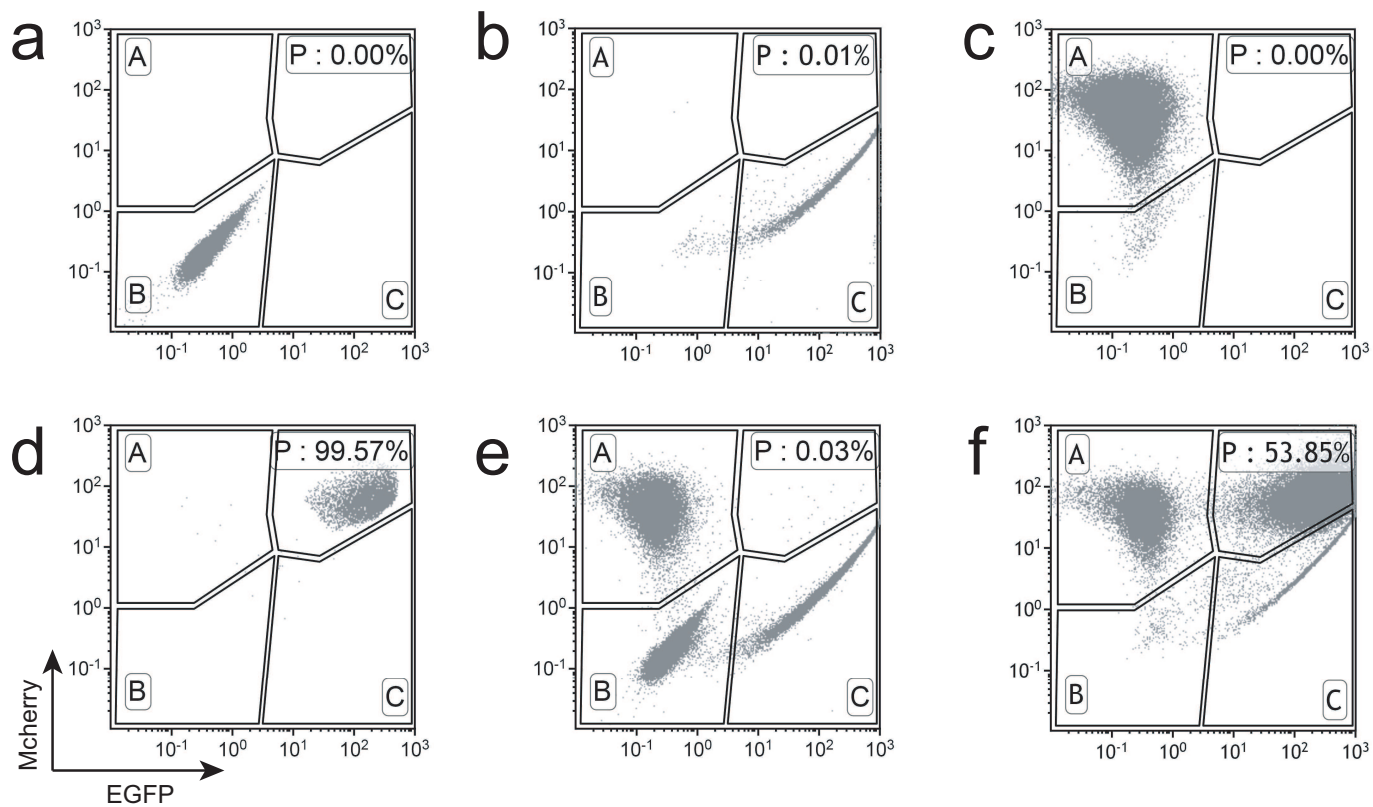

Figure S1: Control gates for FACS

Supplement: Supplementary file 1 — Control gates for FACS. a. Gate B: SKBR3 cells expressing no fluorescent proteins (negative control gate). b. Gate C: SKBR3 cells only expressing EGFP (EGFP control gate). c. Gate A: SKBR3 cells only expressing mCherry (mCherry control gate). d. Gate P: SKBR3 cells expressing both EGFP and mCherry in each cell (positive control gate). e. A mixture of SKBR3 cells expressing no fluorescent protein, EGFP or mCherry. These data were used to calculate the false positive rate. f. A mixture of SKBR3 cells expressing no fluorescent protein, EGFP, mCherry or both EGFP and mCherry. These data were used to calculate the discrimination ability of FACS. (PDF 1136 kb) [file 13277_2015_4337_MOESM1_ESM.pdf]
