# Supplementary material for: Chemotherapy promotes tumour cell hybridization in vivo
Source: Tumour Biol. 2015 Nov 5;37(4):5025–30. doi: 10.1007/s13277-015-4337-7 (PMC4844647; doi:10.1007/s13277-015-4337-7)
Supplement: Supplementary file 2 — (PDF 64 kb) [file 13277_2015_4337_MOESM2_ESM.pdf]

Table S1: Tumour growth rate between chemotherapy and non-chemotherapy groups.

Tumour growth rate(mm^3/day)	5-15 Days	15-18 Days#	18-21 Days
Non-chemotherapy group	216.90±44.44	270.08±59.55	289.32±72.34
Chemotherapy group	167.30±40.69	75.98±39.52	244.37±68.70

#Days of drug injection, $p<0.05$
